# A Cephalometric Comparison of Twin Block and Bionator Appliances in Treatment of Class II Malocclusion

**DOI:** 10.4317/jced.53031

**Published:** 2017-01-01

**Authors:** Fatemeh Ahmadian-Babaki, S. Mehdi Araghbidi-Kashani, Saeedeh Mokhtari

**Affiliations:** 1 Assistant Professor, Department of Pediatric Dentistry, Dental School, Shahid Beheshti University of Medical Sciences, Tehran, Iran; 2 Assistant Professor, Department of Orthodontics, School of Dentistry, Shahed University, Tehran, Iran; 3 Assistant Professor, Department of Pediatric Dentistry, School of Dentistry, Tehran University of Medical Sciences, Tehran, Iran

## Abstract

**Background:**

Class II malocclusion is one of the most common orthodontic problems. In cases of class II malocclusion with mandibular deficiency, functional appliances often are used with the intent of stimulating mandibular growth. Bionator and twin block are two of the more popular functional appliances. The aim of this study was to compare the treatment outcomes of these two appliances using cephalometric radiographs.

**Material and Methods:**

Cephalometric radiographs of 33 patients who had class II division I malocclusion, before and after treatment were digitalized. The mean changes in twin block and bionator groups were compared using independent t test.

**Results:**

Twin block and bionator showed no statistically significant differences in cephalometric parameters except for ANB, NA-Pog, Basal and Ar-Go-Me angles.

**Conclusions:**

There were no statistically significant differences in dentoalveolar and mandibular position between twin block and bionator (*p*>0.1). Twin block was more efficient in inhibition of forward movement of maxilla (*p*<0.1).

** Key words:**Functional, Class II malocclusion, Cephalometrics, Twin block, Bionator, Treatment.

## Introduction

Class II malocclusion is one of the most common orthodontic problems that affects about a third of all subjects seeking orthodontic treatment (1,2). There are several treatment options for the management of this problem; but 2 modalities are used more for 1-phase comprehensive therapy of the malocclusion in the adolescent period: 1-headgear associated with a ﬁxed appliance and Class II elastics; 2-functional jaw orthopedics immediately followed by ﬁxed appliance (2). There are many types of functional appliances (1,3). Maxillary growth alteration, promoted mandibular growth and position, and change in dental and muscular relationships are the expected effects of functional appliances (4).

In cases of class II malocclusion with mandibular deficiency, functional appliances often are used with the intent of stimulating mandibular growth. Many investigators believe the main effect of functional appliances is increasing mandibular growth. In spite some claim that the most significant treatment effects are restricted to dentoalveolar changes (5) because these appliances are supported by teeth, rather than bone (3). So the actual effects of functional appliances remain controversial because studies typically do not distinguish between dental and skeletal components of the correction (3).

Balters’ bionator and twin block are two of the more popular functional appliances; used today. Both of them are tooth-borne but twin block is a full-time wear appliance to use all functional forces applied to the dentition included mastication forces (4,6). There are few studies which have compared the effects of these appliances. The aim of this study was to compare the treatment results of twin block and bionator in patients with cl II malocclusion using their cephalometric radiographs.

## Material and Methods

For this cross sectional study, the dental documents of the patients treated in the clinic of dental school of Shahed University were evaluated. The study protocols were approved by the Regional Committee for Medical Research Ethics. The patients who had following criteria entered the study:

1. Chronologic age of 8-15 years of old

2. Having cl II division I malocclusion 

3. At least end-to- end molar relationship

4. Overjet between 3 and 14 mm

5. Jarabak’s index between 50% and 75 %

6. Complete available treatment documents

7. At least 17 hours appliance wear every day

Also all the subjects should had same bite recording technique including: one step mandibular enhancement, edge-to-edge incisors position and bite opening between 2 to 5 mm and they should be treated using either bionator or twin block.

In addition the subjects who had following criteria were excluded from the study:

1. Maxillary prognatism

2. Severe prognatism of maxillary incisors 

3. Severe dental crowding (space deficiency more than 4 mm)

4. Anterior dental open bite

5. Previous orthodontic treatment

6. Extracted permanent teeth

Finally 33 patients were selected. Among these subjects, 17 patients were treated with twin block and 16 ones were treated with bionator. Average age of patients was 10.3 and 10.9 years old in twin block and bionator group respectively.

Then cephalometric radiographs of subjects, before and after treatment were digitalized using a digital camera (Fuji Film S602, Japan). Afterward the created digital radiographs were analyzed with Onyx Ceph 2.6 software and the changes before and after treatment were defined for each appliance group. The mean changes in twin block and bionator groups were compared using independent t test. *P* value less than 0.1 was considered significant.

## Results

In this study, treatment documents of 33 patients who were treated with twin block or bionator were evaluated. Seventeen subjects (6 females, 11 males) were treated with twin block and 16 patients (7 females, 9 males) had used bionator for their treatment. Mean age of patients at the beginning of the treatment was 10.33 and 10.95 years in twin block and bionator groups respectively.

The changes of cephalometric parameters were analyzed using Jarque-Bera and One-Sample Kolmogorov-Smirnov Tests to evaluate normal distribution of data. As the data showed normal distribution, the independent t test was used to compare the changes between two groups. The results are shown in  Table 1. They revealed that twin block and bionator almost had significant difference in the changes they made in ANB [A point-nasion-B point angle], NA-Pog [Convexity angle], Basal and Gonial angles (*p*< 0.1) and the other cephalometric changes in two groups did not show significant differences.

**Table 1:**
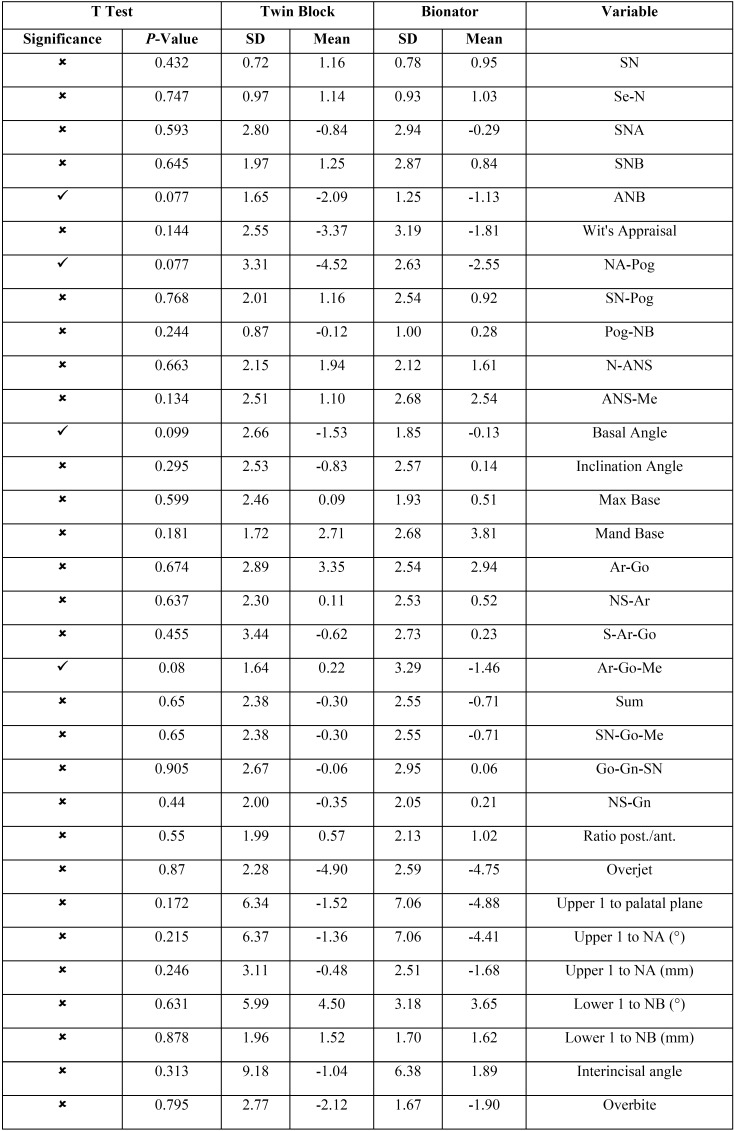
Comparisons of treatment changes between the bionator and twin block groups.

## Discussion

Our aim in this study was to compare the effect of twin block and bionator appliances using cephalometric radiographs. Here we attempted to decrease the individual differences between subjects using inclusion criteria. But the differences in age, sex and pattern of growth always exists. Therefore the differences between twin block and bionator groups do not show pure effect of the type of the appliance and the duration of use. This almost always affects study results.

Skeletal changes

-Maxillary changes: Twin block caused a little more decrease in SNA angle [Sella-Nasion to A Point Angle]. However, the differences between two appliances were not statistically significant. Mc Namara states that SNA angle increases in children during growth; so decrease of this index suggests restriction of forward growth of maxilla during forward posturing of the mandible. The functional appliances leave reciprocal force acting distally on the maxilla (headgear effect). This would be ideal for correction of a CLII skeletal discrepancy (7). But the two appliances do not actually restrict maxillary growth because maxillary length in both groups increased after treatment however this increase was less in twin block group. SNA angle decrease can also be explained by the changes of maxillary plane angle (inclination angle). This index was decreased in twin block group and increased in bionator group. The two appliances did not differ significantly but this can probably support more headgear effect of twin block. This agrees with some studies (4,7-10) but others believe that the design of the appliance is not major factor in the headgear effect of functional appliance therapy (4,11-14).

-Mandibular changes: Our results show that mandibular length changes were greater in bionator group and this finding is consistent with the result of many investigators (15); however others report more mandibular length changes in twin block group (4). Effective mandibular length assessment should be discriminated considering mandibular length and ramus height (Ar-Co). Evaluating Ar-Co distance, patients using twin block showed more ramus height increase. However the difference between two appliances was not statistically significant. Illing (1998) used Cd-Gn index [Mandibular Length; distance between posterior border of mandibular condyle and Pogonion] to evaluate the mandibular changes and states that bionator resulted in more changes than twin block but there was no statistically difference (8).

The two appliances resulted in NA-Pog angle decrease but the twin block had statistically greater effect. It probably means that twin block results in more forward positioning of Pog [Pogonion; Most anterior point of mandibular symphysis] (and less forward positioning of A point. So twin block is more effective in improving patient’s profile. Changes of the facial angle [SN-Pog index] also confirms the greater forward positioning of Pog point with twin block; however the two appliances were not statistically different when comparing the facial angle. Illing also agrees with our findings (8).

-Maxillomandibular relation changes: Anteroposterior relationship: In both groups, ANB angle was decreased after treatment but twin block resulted in more decrease and this was statistically significant. Limited anterior movement of A point or anterior movement of B point may be the cause of ANB angle decrease. Wit’s appraisal index also confirms these findings; however this index did not differ significantly between two appliances. Jena and Illing also state that twin block has more effect on anterior-posterior relationship of maxilla and mandible (4,8).

-Vertical changes: Righellic and Mc Namara *et al.* reported that functional appliances do not change the craniofacial growth pattern however Nielsen states that facial height has been increased (8). In our study, the height of lower facial third was increased in both groups. However there was no statistically significant difference between two appliances, bionator resulted in more increase. This is explained by the Clockwise rotation of mandible after using functional appliances. The changes of y axis [The angle of Sella-Nasion(SN) to Sella-Gnathion(S-Gn)] and GO-Gn-SN [The angle of SN to Gonion-Gnathion] confirms the increase of lower facial height but this is not in consistent with changes of gonial angle [Ar-Go-Me: Articulare (Ar)- Gonion(Go)- Menton (Me) angle] and SN-Go-Me [The angle of SN to Gonion-Menton] which shows gonial angle decrease. This can be explained by the increase of ramus height and also biases of the study, the effect of other cephalometric indices and different individual growth patterns.

Changes of upper facial height (N-ANS [Anterior Nasal Spine]) were less in bionator group however the two appliances did not differ significantly. Probably the headgear effect of functional appliances is responsible for the changes of ANS [Anterior Nasal Spine] and therefor the upper facial height.

Dental changes.

Changes of overjet are the result of both skeletal and dentoalveolar effects of the treatment. It means mandibular advancement and also changes of upper and lower incisor inclination affect overjet. So overjet is not a pure indicator of dentoalveolar changes. But dental changes can be assessed as a factor to distinguish the skeletal or dental effects of functional appliances. In our study Patients who used Twin block had greater overjet decrease. But there was no significant difference between two groups. Jena and Illing also confirm our finding (4,8).

Both bionator and twin block appliances result in lingual tipping of the upper incisors. This is also shown by many other investigators for almost all functional appliances (16-18) but upper 1 to palatal plane angle was decreased more in bionator group. Upper 1 to palatal plane angle change is an indicator of dentoalveolar effect of the appliance. Dentoalveolar changes reduce the growth modification and skeletal effects of functional appliances. So it is not favorable. Overall it can be assumed that bionator result in a little more dentoalveolar changes of upper incisors, in this investigation.

Evaluating the mandibular incisors and NB [Nasion to B Point] reveals that subject using twin block had more inclination of lower incisors; but linear distance changes of lower incisors and NB shows that bionator results in more variations in mandibular incisor positions. However the two groups did not differ significantly from this point. This was similar with the findings of Jena and Illing (4,8). The proclination of the lower incisors is probably consequent to the resultant mesial force on the lower incisors induced by the protrusion of the mandible (8).

In conclusion, in our study:

1. Twin block and bionator didn’t have significant difference in the changes they made in most cephalometric parameters except ANB, NA-Pog, Basal and Gonial angles.

2. Twin block and bionator did not differ significantly in the dental changes they made.

3. Mandibular growth enhancement was almost similar in two groups.

4. The two appliances had significant different effect on NA-Pog index and twin block has better effect on the correction of profile convexity.

5. Twin block resulted in greater changes of ANB angle. This probably means this appliance causes more improvement in antero-posterior relation.

6. Probably the significant difference of twin block and bionator in the three parameters including basal angle, NA-Pog and ANB angle reveals the greater headgear effect of twin block. It means this appliance has more inhibition effect on forward displacement of A point and forward growth of maxillary plane.
